# Suppression of MRI Truncation Artifacts Using Total Variation Constrained Data Extrapolation

**DOI:** 10.1155/2008/184123

**Published:** 2008-09-07

**Authors:** Kai Tobias Block, Martin Uecker, Jens Frahm

**Affiliations:** Biomedizinische NMR Forschungs GmbH, Max-Planck-Institut für biophysikalische Chemie, 37070 Göttingen, Germany

## Abstract

The finite sampling of *k*-space in MRI causes spurious image artifacts, known as Gibbs ringing, which result from signal truncation at the border of *k*-space. The effect is especially visible for acquisitions at low resolution and commonly reduced by filtering at the expense of image blurring. The present work demonstrates that the simple assumption of a piecewise-constant object can be exploited to extrapolate the data in *k*-space beyond the measured part. The method allows for a significant reduction of truncation artifacts without compromising resolution. The assumption translates into a total variation minimization problem, which can be solved with a nonlinear optimization algorithm. In the presence of substantial noise, a modified approach offers edge-preserving denoising by allowing for slight deviations from the measured data in addition to supplementing data. The effectiveness of these methods is demonstrated with simulations as well as experimental data for a phantom and human brain in vivo.

## 1. INTRODUCTION

In MRI, spatial information is obtained from the object using magnetic field gradients, which link the Larmor frequency of the excited spins to their spatial location. Thus, the received signal is the continuous Fourier transform of the object's proton density

(1) $$S(\overset{\to }{k})\;=\int \rho (\overset{\to }{x})\;e^{-i\overset{\to }{k}\cdot \overset{\to }{x}}\;d\overset{\to }{x}\;,$$

where the *k*-space position $\overset{\to }{k}$ can be calculated from the time course of the applied gradients. In practice, the proton density is further modulated by spin relaxation, off-resonance effects, and other mechanisms all neglected here.

It is well known that objects with compact support have a Fourier transform with nonlimited support. For example, the Fourier transform of a rectangle is composed of sinc functions in each dimension. Because only a single location of the Fourier space can be measured at a time, it is impossible to fully sample such Fourier transform by travelling the MRI *k*-space with magnetic field gradients. Hence, there are two experimental restrictions for MRI. First, the continuous Fourier transform is sampled discretely, which can be seen as a multiplication with a comb-function in frequency space. In image space, this corresponds to a convolution with a reciprocally spaced comb-function and leads to periodic object copies with a spacing inverse to the sample distance in *k*-space. Second, the Fourier transform is sampled only within a finite region around the *k*-space center with all other information missing.

In the conventional case, a discrete Fourier transformation of the finitely measured data is performed to reconstruct an image. This strategy implicitly assumes that the Fourier transform is zero everywhere outside the sampled region. It is clear that the assumption is not very appropriate for finite objects, although the corresponding reconstruction totally complies with all data measured. In fact, any solution that coincides at the sampling positions is a valid reconstruction, because the finite sampling pattern opens degrees of freedom from the null space of the projection evoked by finite sampling. Setting this null space to zero is a simple and convenient solution. Unfortunately, however, the procedure corresponds to a multiplication of the true object's Fourier transform with a rect-function (in case of Cartesian sampling) which, in image space, results in a convolution of the true object with a sinc-function. This effect is well known as truncation artifact or Gibbs ringing and mainly appears as an oscillating overshoot of the image intensity near discontinuities [1, 2]. Although the problem may be reduced by increasing the measured *k*-space, many practical applications still rely on acquisitions with a relatively low-matrix resolution in at least one image dimension, and therefore suffer from respective artifacts.

So far, various methods have been developed to ameliorate image disturbances due to finite sampling [2–5]. However, in the majority of MRI applications and, in particular, for most commercially available MRI systems, only a simple data filtering is routinely employed. In this case, visual reduction of the ringing artifacts is achieved by a smearing of the intensity oscillations, which leads to an undesired loss of image resolution. Alternative methods attempt to extrapolate the measured data and thereby avoid a sharp cut-off in *k*-space [6–9]. A key difference to the filtering approach is that the actually measured data is not changed but supplemented with synthetic data—a reasonable strategy as the measured data is not incorrect but only incomplete. This can be achieved by exploiting a priori knowledge about the true object and, consequently, all extrapolation techniques rely on certain assumptions, where the existing methods follow different strategies. In this regard, the present work demonstrates that also the very unspecific assumption of a piecewise-constant object can be utilized to successfully extrapolate data in *k*-space and concomitantly reduce the ringing artifacts without compromising image resolution.

## 2. THEORY

**Figure 1:**
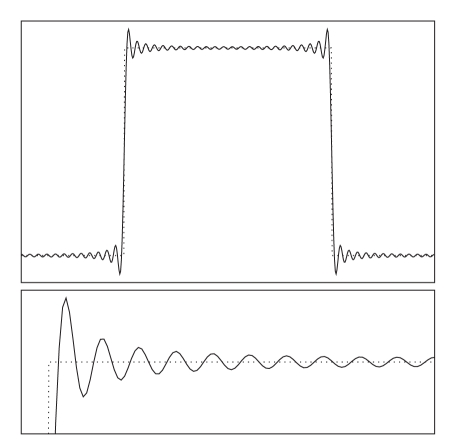
(Top) One-dimensional profile of a rectangle reconstructed by Fourier transformation from 96 Fourier samples (solid line) in comparison to the true function (dotted). While the true function is piecewise constant, the Fourier reconstruction exhibits severe ringing artifacts due to truncation of the Fourier coefficients, which causes an increased total variation (TV) value. (Bottom) Magnified view.

Figure 1 compares the one-dimensional profile of a rectangle reconstructed by Fourier transformation from only 96 Fourier samples to that of the original function. It clearly illustrates severe ringing artifacts, although the true function is piecewise constant and free of any oscillations. Such oscillations can be quantified using the total variation (TV), which sums the modulus of jumps between all neighboring pixels of a reconstructed image *I*(*x*,*y*)

(2) $$TV(I)\;=\overset{N}{\underset{y=0}{\sum }}\;\overset{N}{\underset{x=0}{\sum }}|I(x,\;y)-I(x-1,\;y)|\;+\;|I(x,\;y)-I(x,\;y-1)|.$$

The TV concept was initially introduced to image processing by Rudin et al. [10] for denoising applications because noise patterns create a high TV value relative to that of a noise-free image, and they become particularly reduced when modifying the image in such a way that the TV value is minimized. As a specific property, edges are preserved during this procedure, and thus TV minimization emerged as one the most popular denoising techniques. In recent years, the TV concept is attracting strong interest in the field of compressed sensing [11] because for specific sampling techniques, the TV value can be utilized to identify and to remove artifacts from undersampling, offering a remarkable reduction of the measurement time [12]. In a similar manner, truncation artifacts lead to an increased TV value relative to that of the true object, so that the TV may also be taken as a measure of the artifact strength for finite *k*-space sampling, which has been recognized by Landi et al. as well [13]. Therefore, the proposed idea is to add a set of synthetic frequencies $\vec{v}$ to the measured data $\vec{y}$, which is specifically chosen such that the TV value of the image reconstructed from the combination of the measured and synthetic data is minimized

(3) $$\overset{\to }{v}\;=\;\underset{\overset{\to }{v}}{\text{arg}\text{min}}\;\;\text{TV}(ℱ\{\overset{\to }{v}⊕\overset{\to }{y}\})\;,$$

where *ℱ* denotes the discrete Fourier transformation. Interestingly, by searching for the set of synthetic frequencies $\vec{v}$, the unmeasured *k*-space data is recovered to a certain degree if the assumption of a piecewise-constant object is appropriate.

Estimation of the synthetic data can be achieved by minimizing (3) with a nonlinear numerical optimization technique. The present proof-of-principle implementation used the CG-Descent algorithm [14], which is a recent variant of the nonlinear conjugate gradient method that allows to rather efficiently solve large-scale problems. The algorithm can be used in a black-box manner, requiring only the evaluation of a cost function and its gradient for given estimate vectors $\vec{v}$. The cost function is needed to quantify the goodness of a given estimate (i.e., it is small for a good estimate and large otherwise), and for the problem defined in (3) it simply has the form

(4) $$\Phi (\overset{\to }{v})\;=\;\text{TV}(ℱ\{\overset{\to }{v}⊕\overset{\to }{y}\})\;.$$

The gradient of the cost function corresponds to the derivative of this function with respect to all components of the estimate vector $\vec{v}$. Because the discrete Fourier transformation is a unitary operation, it can be evaluated conveniently by calculating the gradient of the TV term in the image domain (i.e., estimating a vector that describes how the TV value changes for modifications of the individual pixels), followed by an inverse Fourier transformation to the frequency domain.

### 2.1. Extended TV formulation

Calculation of the TV value according to (2) uses only the first-order derivative of the image with respect to its *x*- and *y*-directions. This value is minimized if an image consists of areas with constant signal intensity, so that the extrapolation procedure yields a solution primarily with constant areas. While desirable for truly flat objects like numerical phantoms, it tends to create images with a slightly blocky or patchy appearance for real-world objects. Therefore, it is advisable to additionally include second-order derivatives into the TV term, which then allows for intensity gradients in the images and yields more naturally looking solutions

(5) $$\begin{array}{ll} & \text{T}{\text{V}}_{2}(I)\;=\\ & \;\;\overset{N}{\underset{y=0}{\sum }}\;\;\overset{N}{\underset{x=0}{\sum }}\;\sigma \cdot (|I(x,\;y)-I(x-1,\;y)|\;+\;|I(x,\;y)-I(x,\;y-1)|)\\ & \;\;\;\;\;\,\;\;\;\;+(1-\sigma )\cdot \left(|I(x-1,\;y)-2\cdot I(x,\;y)\;+\;I(x\;+\;1,\;y)\right|\\ & \;\;\;\;\;\;\;\;+|I(x,\;y-1)-2\cdot I(x,\;y)\;+\;I(x,\;y\;+\;1)|\\ & \;\;\;\;\;\;\;\;+|I(x,\;y)-I(x-1,\;y)-I(x,\;y-1)\\ & \;\;\;\;\;\;\;\;\;+I(x-1,\;y-1)|). \end{array}$$

Here, $\sigma \in [\begin{array}{cc} 0 & 1 \end{array}]$ is a weighting factor which can be used to tune the images between a slightly more blocky looking and a slightly smoother appearance. For the reconstructions presented, it was set to *σ* = 0.77 based on the considerations by Geman and Yang [15].

### 2.2. Edge-preserving denoising

In practice, experimental MRI data can be significantly contaminated by Gaussian noise. While the aforementioned approach is still able to reduce visible truncation artifacts under these circumstances, it does not reduce image noise because the measured *k*-space data remains unchanged. On the other hand, an additional denoising may be achieved by loosing the fixed bound on the measured data, that is by introducing a data fitting term. In this case, the algorithm not only adds synthetic frequencies to obtain a TV minimization, but is also allowed to find a solution that slightly diverges from the measured data, which yields an effective edge-preserving denoising. Therefore, the estimate vector $\vec{v}$ has to be extended such that it contains both synthesized frequencies as well as frequencies from the measured part of *k*-space, which is indicated by writing ${\vec{v}}_{d}$ instead.

In the denoising case, the cost function takes the form

(6) $$\Phi ({\overset{\to }{v}}_{d})\;=\;\lambda \cdot ∥{\overset{\to }{v}}_{d}⊖\overset{\to }{y}{∥}_{2}^{2}\;+\;\text{TV}(ℱ\{{\overset{\to }{v}}_{d}\})\;,$$

where ⊖ denotes an operation that calculates the residual between the measured values and the corresponding entries of the estimate, which are now contained in the vector ${\vec{v}}_{d}$. Further, *λ* is a weighting factor that allows to select the desired denoising strength. While a low weight permits considerable divergences from the measured values and, thus, leads to an effective removal of noise, it can also cause a loss of object detail if selected too low. Therefore, the weight has to be adjusted with respect to the signal-to-noise ratio of the measurement sequence, where a reasonable strategy is to estimate a fixed value once for each protocol by computing a set of test images with different *λ* values and selecting the value yielding the desired degree of denoising.

### 2.3. Phase variations

Although the basic physical quantity measured by MRI, that is, the spin-density modulated by relaxation or saturation effects, should be real-valued and nonnegative in theory, inherent experimental phase variations usually cause the observed object to be complex-valued. Moreover, modern MRI systems often use multiple receive coils with complex-valued sensitivity profiles, yielding differently modulated views of the object. As a consequence, spatially varying transitions between the real and imaginary component occur as well as local intensity changes, which conflict with the assumption of a piecewise-constant quantity and prevent a direct application of the TV constraint. Therefore, some mechanism is required to cope with the phase variations and the multicoil scenario.

In this proof-of-principle study, phase variations were removed in a preprocessing step by performing a Fourier transformation of the data from each coil and calculating the sum-of-squares of all channels in the image domain. Subsequently, an inverse Fourier transformation of the sum-of-squares data was performed to obtain a combined data set with real-valued and nonnegative values in the image domain, which enables a calculation of the TV value using only the real part of the image. While this simple technique turned out to be sufficient for demonstrating a removal of truncation artifacts by TV-constrained data extrapolation, routine applications will probably require a more sophisticated procedure, in particular when combined with advanced techniques such as parallel imaging and when using complex coil configurations with more localized sensitivities of the individual receiver elements.

## 3. METHODS

Simulations were performed with the Shepp-Logan phantom, which is composed of several ellipses. Because the Fourier transform of a single ellipse is given by a Bessel function, an analytical Fourier transform of the phantom is obtained by a superposition of respective Bessel functions. Truncation artifacts can be studied by evaluating the noncompact analytical transform at the sampling positions along the trajectory, here yielding a matrix of 96 × 96 Fourier samples. All simulations and processing of experimental data were done offline using an in-house software package written in C/C++.

MRI experiments were conducted at 2.9 T (Siemens Magnetom TIM Trio, Erlangen, Germany) with use of a receive only 12-channel head coil equipped with hardware signal combiners, yielding four receiver channels with different combinations of the coil elements. Measurements were performed for a water phantom and human brain in vivo, where written informed consent was obtained from all subjects prior to each examination. For demonstration purposes, the image acquisitions were done with a simple slice-selective spin-echo sequence at a 200 × 200 mm^2^ field of view, covered by a 96 × 96 acquisition matrix. Different sequence settings were used to obtain data sets with low and high level of noise, where the latter was achieved by reducing the flip angle and slice thickness while increasing the receiver bandwidth. Further, one data set was acquired with a slice-selective gradient-echo sequence, which allowed for the rapid measurement of a full 288 × 288 acquisition matrix.

All images were reconstructed on a 288 × 288 matrix corresponding to an extrapolation factor of 3. The proposed algorithm was run for a fixed number of 3000 iterations, which takes about 2-3 minutes on a standard microprocessor. In cases where an additional data fitting term was used, the weighting factor *λ* was adjusted manually to yield a reasonable solution as judged by visual inspection. Zero-padded solutions with and without filtering were calculated for comparison. Here, a simple Lanczos sigma filter, that is, multiplication with a sinc-function, was applied, where the window width was selected such that the sinc-function's first null coincides with the border of the measured *k*-space. Although other filters might perform better, it serves to demonstrate the general problem related to data filtering. In addition, a two-dimensional version of the extrapolation method described by Constable and Henkelman [6] was implemented with a window width of *P* = 2 for the edge-preserving sigma filter. In our implementation, the filter parameter Δ was selected according to Δ = *c* · *I*, where *I* denotes the intensity of the pixel to be filtered and *c* is a global coefficient that was set to *c* = 0.1 based on visual inspection. Finally, all images were magnified and cropped to improve the visibility of the artifacts.

## 4. RESULTS

**Figure 2:**
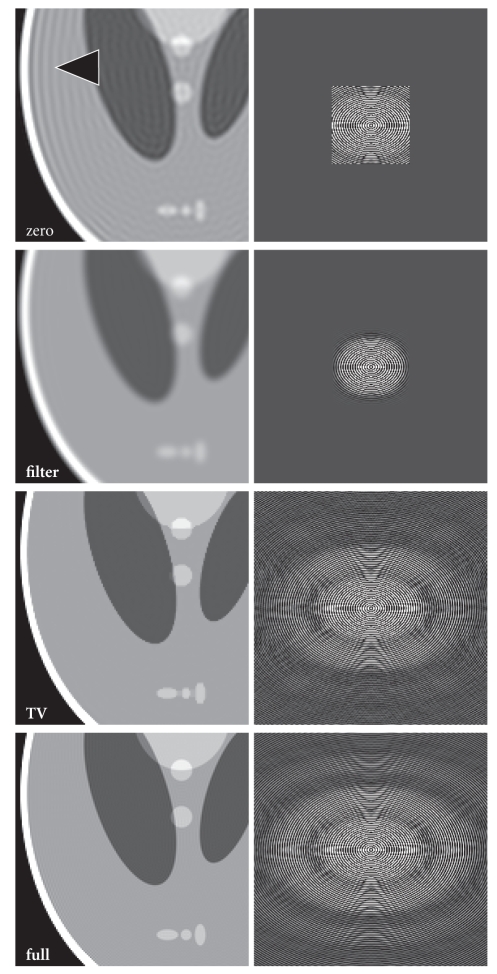
(Left) Images of the numerical Shepp-Logan phantom (96 × 96 samples, 288 × 288 reconstruction matrix) and (right) corresponding *k*-space representations reconstructed using zero-padding (zero), filtered zero-padding (filter), and the proposed extrapolation method (TV). For comparison, a data set with a fully sampled 288 × 288 matrix is shown (full). Arrow = truncation artifact.

Figure 2 shows different reconstructions of the Shepp-Logan phantom (left column) together with the respective Fourier transforms (right column). It is clearly visible that the zero-padded solution (zero) suffers from severe ringing artifacts around all edges of the phantom. The extent of the measured *k*-space can be seen in its Fourier transform. Most ringing artifacts disappear after filtering (filter), however, at the expense of a significant loss of image resolution. In contrast, the image reconstructed with the proposed method (TV) is neither affected by ringing artifacts nor by blurring, and it presents with considerably sharper edges relative to the zero-padded solution. Its Fourier transform reveals that the measured data has been properly extrapolated into the uncovered areas of *k*-space. For comparison, a full reconstruction from 288 × 288 samples is shown in the bottom row (full).

**Figure 3:**
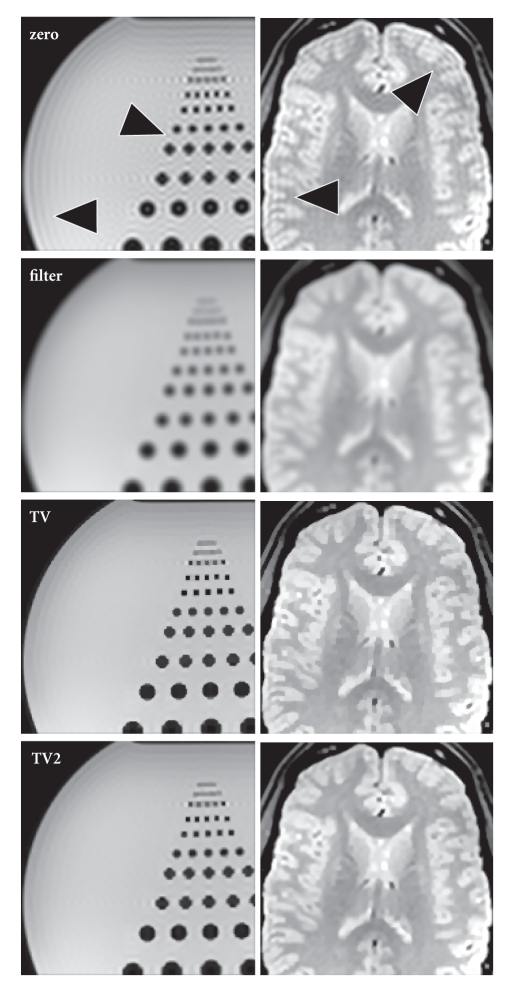
Spin-echo images (96 × 96 samples, 288 × 288 reconstruction matrix) of (left) a phantom (TR/TE = 4000/8 ms, BW 243 Hz/pixel, FA 70°, 3 mm slice) and (right) a human brain in vivo (TR/TE = 4000/25 ms, BW 180 Hz/pixel, FA 90°, 2 mm slice) using zero-padding (zero), filtered zero-padding (filter), the proposed extrapolation method with first-order (TV), and additionally second-order derivatives (TV2). Arrows = truncation artifacts.

Figure 3 demonstrates the application of the method to experimental data obtained for a phantom (left column) and a human brain in vivo (right column) in comparison to zero-padded (zero) and filtered zero-padded solutions (filter). Again, the ringing artifacts obtained for zero padding (indicated by arrows) are significantly reduced when using TV-constrained data extrapolation with only first-order (TV) or additionally second-order derivatives (TV2). The blocky appearance of the TV reconstruction becomes much more smoother for the TV2 approach, although both solutions (TV and TV2) look somewhat more blocky than the zero-padding solution.

**Figure 4:**
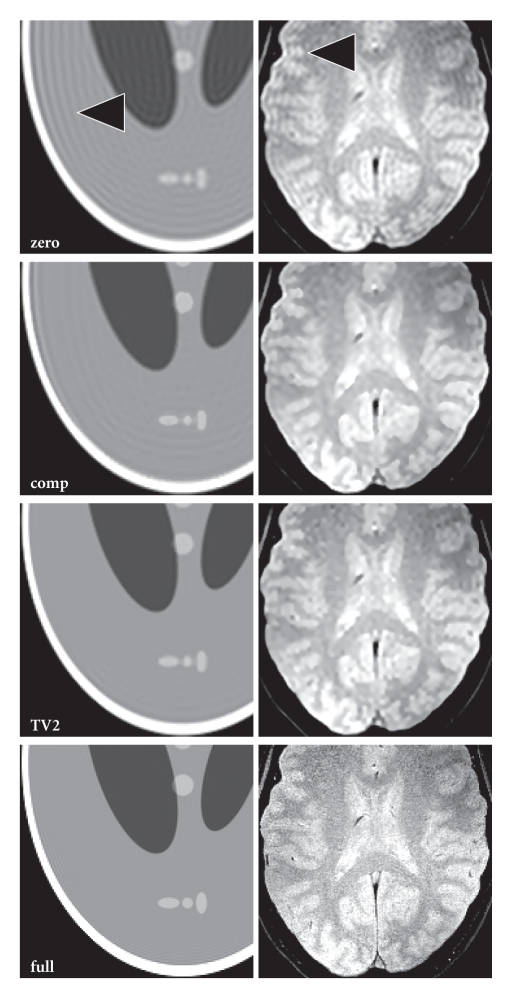
(Left) Images of the Shepp-Logan phantom (96 × 96 samples, 288 × 288 reconstruction matrix) and (right) gradient-echo images of the human brain in vivo (TR/TE = 500/8 ms, BW 80 Hz/pixel, FA 30°, 2 mm slice) reconstructed using zero-padding (zero), the extrapolation method by Constable and Henkelman (comp), and the proposed method with first- and second-order derivatives (TV2). For comparison, reconstructions from fully sampled 288 × 288 matrices are shown (full). Arrows = truncation artifacts.

In Figure 4, the proposed approach (TV2) is compared to a 2D version of the extrapolation method by Constable and Henkelman (comp) for the Shepp-Logan phantom (left column) and an experimental study of the human brain in vivo (right column). It can be seen that the performance of the proposed extrapolation method is slightly better for the simulated data, while both approaches yield an effective suppression of ringing artifacts for the experimental data (note that the alternative method leads to a slight denoising of the image). However, in our implementation, the algorithm by Constable and Henkelman tends to be sensitive to the parameter selection for the initial filter that is used to detect true edges of the object, whereas a selection of respective parameters is not needed in the TV-based approach.

**Figure 5:**
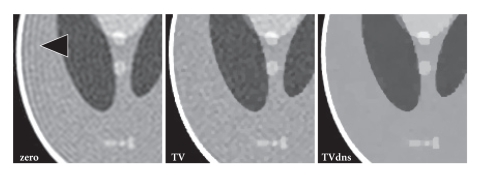
Images of the numerical Shepp-Logan phantom reconstructed from noisy data (96 × 96 samples, 288 × 288 reconstruction matrix) using zero-padding (zero), the proposed extrapolation method (TV), and the proposed method combined with denoising (TVdns). Arrow = truncation artifact.

Figure 5 shows reconstructions of the Shepp-Logan phantom from noisy data using zero-padding (zero), the proposed extrapolation approach (TV), and its combination with denoising (TVdns). While the basic extrapolation approach leads to a reduction of truncation artifacts also for noisy data, it does not reduce the noise patterns. However, when extending the TV penalty to the measured data, the method effectively flattens noise patterns in addition to the suppression of ringing artifacts.

**Figure 6:**
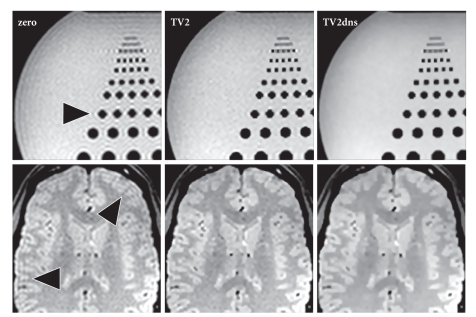
Spin-echo images of (top) a phantom (TR/TE = 4000/100 ms, BW 789 Hz/pixel, FA 50°, 1 mm slice) and (bottom) a human brain in vivo (TR/TE = 4000/15 ms, BW 401 Hz/pixel, FA 70°, 1 mm slice) reconstructed from noisy data (96 × 96 samples, 288 × 288 reconstruction matrix) using zero-padding (zero), the proposed extrapolation method (TV2), and the proposed method combined with denoising (TV2dns). Arrows = truncation artifacts.

Finally, corresponding reconstructions from experimental data with a high degree of noise are shown in Figure 6. Here, a combination of first- and second-order derivatives was used for the TV calculation. As in the simulations, the proposed method leads to a reduction of truncation artifacts (TV2), while the extension to data fitting yields an additional edge-preserving denoising (TV2dns).

## 5. DISCUSSION

### 5.1. Accuracy and limitations

Both simulations and experiments demonstrate that TV-constrained data extrapolation effectively reduces truncation artifacts due to finitely sampled MRI acquisitions. Usually, the images exhibit a more blocky appearance compared to zero-padding. However, it should be noted that the smoothness observed for zero-padding originates to a significant degree from the convolution with the sinc-function. As a consequence, a sharp edge of the object is mapped as a rather smooth pattern, which might appear more familiar to the viewer than a blocky image, but strictly represents an image artifact. Hence, the extrapolation technique may even lead to a slight gain of resolution due to a sharpening of the point-spread function, following from the reciprocity property of the Fourier transformation. This effect can be best seen in Figure 6 when comparing the borders of the dark brain vessels obtained for zero padding (zero) with the proposed method (TV2).

Residual image artifacts are explained by multiple reasons. First, the method is based on the assumption that the true object is piecewise constant, which is only approximately valid for real-world objects. In the presence of additional experimental effects like flow artifacts, the assumption might be even less appropriate. Hence, the extrapolation performance depends on the object's conformance with the assumption that it is piecewise-constant. Moreover, if the true object contains strongly varying patterns, the algorithm may erroneously soften such patterns by supplementing respective high frequencies. On the other hand, in the majority of cases, the assumption of a piecewise-constant object seems to be more appropriate than that of all conventional reconstructions, namely, a Fourier transform of the object that is zero outside the sampled *k*-space area.

Second, the proposed method synthesizes only a finite number of additional frequencies, whereas an infinite number of *k*-space samples would be required to completely eliminate all truncation effects. In practice, however, it turned out that there is no perceivable benefit of extrapolating by a factor of higher than three. The reason is that the method yields an implicit filtering of the extrapolated data: assuming that the extrapolation procedure would recover the unmeasured *k*-space samples exactly, then a new truncation effect would arise at the extended border and again lead to ringing artifacts in image space (though with a higher oscillation frequency). Because this would increment the TV value, the method automatically lowers outer frequencies during the extrapolation procedure to prevent the upcoming of new ringing artifacts. Hence, the extrapolated values diverge categorically from the true frequencies which, in this case, is a rather desirable feature as the prime target is to reduce visually annoying ringing artifacts rather than to gain super-resolution.

Third, if a completely artifact-free reconstruction of the object would be available, then respective frequency samples could be calculated with a discrete Fourier transformation of the given image. Interestingly, these samples would diverge from the experimentally measured frequencies, because image pixels are discrete and, thus, the Fourier transform of the image is periodic such that outer frequencies from neighboring copies (of the true object's noncompact Fourier transform) overlap. This is different from the experimental situation where the object is continuous and the outer frequencies are missing instead of overlapping. Consequently, an artifact-free discrete reconstruction can only be obtained if the samples used for the reconstruction specifically diverge from the measured frequencies. A complete artifact removal, therefore, requires to alter the measured frequencies instead of keeping them unchanged. Unfortunately, the information how the samples have to be adjusted is not available, so that in practice a data fitting term might be the best solution when a complete removal of ringing artifacts is needed. However, this might cause a loss of object detail as described in the theory section.

### 5.2. Implementation issues

The modulus function in the TV formula (2) has a fundamental role for the success of TV-based image processing. Because subtraction of neighboring pixels—performed before taking the modulus—can be seen as applying a difference operator to the estimate, TV minimization yields a solution with minimum L1-norm in the difference basis. Due to the specific character of the modulus function, this solution tends to be sparse in the difference basis: it has few large jumps and most differences between neighboring pixels are near zero, which directly translates into a piecewise-constant image (and explains the edge preserving character of TV-based denoising). If the modulus would be replaced by a square function, then the optimizer would try to find a minimum L2-norm solution with minimal jumps between all neighboring pixels. This corresponds to a globally smooth image, which is usually undesired due to a loss of sharp edges. While it is rather simple to obtain a minimum L2-norm solution as its cost function is strictly convex, finding a minimum L1-norm solution is much more challenging; and many optimization algorithms fail if directly applied to the TV problem. One major reason is that the derivative of the modulus function is just ± 1, which does not help to guess a reasonable step size toward the function's minimum. However, it turned out that the CG-Descent algorithm is capable to handle the problem as it comprises a powerful line-search procedure, but it is probably not the optimal method for finding the solution. In particular, the convergence tends to be somewhat sensitive to the scaling of the data. In order to ensure convergence, it was, therefore, necessary to introduce a scaling factor that limits the modification strength for each iteration and to run the algorithm in turn for a high number of iterations (e.g., 3000 iterations as arbitrarily chosen here). Nevertheless, this issue should not be seen as a drawback of the proposed extrapolation approach itself, but rather as a technical aspect of the optimization method utilized in this proof-of-principle study. Employing a dedicated algorithm for TV minimization should render a scaling factor unnecessary and significantly improve the convergence rate. Although such enterprise promises reconstructions in a fraction of the current processing time, it is outside the scope of the present study.

## 6. CONCLUSION

The present work demonstrates that the simple assumption of a piecewise-constant object can be exploited to extrapolate measured data in *k*-space. This allows for a significant reduction of ringing artifacts that arise from data truncation in *k*-space. In contrast to commonly used filtering approaches, the method does not degrade the spatial resolution of the reconstructed image and rather leads to a mild resolution enhancement due to sharpening of the point-spread function. If the measured data is seriously contaminated by noise, an extended approach offers edge-preserving denoising by slightly altering the measured data in addition to supplementing synthetic data. Both variants can be implemented as a pure postprocessing procedure and are also applicable for partial Fourier acquisitions. Therefore, no modification of the MRI sequence is required. While the current implementation suffers from a relatively high computational load, the use of a dedicated TV optimization algorithm promises a processing speed suitable for routine applications.
